# Flexible Fe_3_O_4_/Ag/RGO Triple-Layer-Coated Cotton Fabric for Electromagnetic Interference Shielding

**DOI:** 10.3390/polym18091035

**Published:** 2026-04-24

**Authors:** Houqiang Hua, Shulan Xiang, Ronghui Guo

**Affiliations:** 1 College of Aviation Electronic and Electrical Engineering, Civil Aviation Flight University of China, Chengdu 641419, China; huahouqiang@cafuc.edu.cn; 2 College of Flight Technology, Civil Aviation Flight University of China, Guanghan 618307, China; 3 College of Biomass Science and Engineering, Sichuan University, Chengdu 610065, China; ronghuiguo214@126.com

**Keywords:** Fe_3_O_4_/Ag/RGO-coated, cotton fabric, electromagnetic shielding, electrical conductivity

## Abstract

With the rapid development of electronic devices and wireless communication systems, electromagnetic interference pollution has become a critical concern, driving the urgent demand for high-performance, lightweight, and flexible electromagnetic interference (EMI) shielding materials. To endow fabrics with excellent electromagnetic shielding, a Fe_3_O_4_/Ag/RGO ternary nanocomposite-coated cotton fabric for electrical conductivity and EMI shielding application was developed. The cotton fabric pretreated with dopamine was coated with graphene oxide (GO), followed by silver nanoparticles (Ag) via a microwave-assisted chemical reduction method, and Ag/reduced graphene oxide (RGO)-coated cotton. Subsequently, nano-ferroferric oxide was deposited on Ag/RGO-coated cotton fabric using a coprecipitation method. The results show that the surface resistance of Fe_3_O_4_/Ag/RGO-coated cotton fabric arrives at 1.68 Ω/sq, demonstrating excellent electrically conductive performance. Fe_3_O_4_/Ag/RGO-coated cotton fabric demonstrates outstanding electromagnetic shielding performance, with SE values exceeding 45 dB across the entire 1–18 GHz range. The flexibility and superior electromagnetic shielding performance of Fe_3_O_4_/Ag/RGO-coated cotton fabric render it a promising candidate for applications in wearable electronics, aerospace, advanced protective systems, and military protective clothing.

## 1. Introduction

In recent decades, the rapid proliferation of portable electronic devices and wireless communication technologies has led to a significant increase in electromagnetic (EM) radiation pollution. This unintended byproduct of technological advancement poses serious threats to the precision of sensitive electronic equipment and has been linked to potential adverse effects on human health [1,2]. Consequently, the development of high-performance electromagnetic interference (EMI) shielding materials has become a critical area of research. Traditionally, metals such as copper and aluminum have been employed as shielding materials due to their high electrical conductivity [3,4]. However, their practical applications are often constrained by drawbacks including high density, susceptibility to corrosion, poor flexibility, and complex processing requirements. To be more specific, metal-based shields typically exhibit a reflection-dominated shielding mechanism, which generates secondary electromagnetic pollution that is detrimental to nearby electronic devices and potentially harmful to human health. Moreover, their high density makes them unsuitable for lightweight wearable and portable applications, while their inherent rigidity limits integration into flexible textile platforms. These limitations have driven the search for lightweight, flexible, and efficient alternatives, with textile-based materials emerging as promising candidates due to their inherent flexibility, lightweight nature, and potential for integration into wearable technologies.

Among various textile substrates, cotton fabric has attracted considerable attention due to its low cost, excellent flexibility, biocompatibility, and porous structure, which facilitates the incorporation of functional nanoparticles [4,5,6]. To transform insulating cotton fabrics into conductive and EMI shielding materials, a common strategy involves depositing conductive nanomaterials onto the fabric surface. Graphene and its derivatives, particularly reduced graphene oxide (RGO), have been widely investigated for this purpose [7]. RGO possesses a unique two-dimensional structure with exceptional electrical conductivity, large specific surface area, and high mechanical strength [2,7]. Nevertheless, the pristine RGO-coated fabrics often suffer from limited electrical conductivity because an incomplete reduction in graphene oxide leaves residual oxygen-containing functional groups that disrupt the conjugated sp^2^ carbon network. In addition, RGO provides only dielectric loss without any magnetic loss contribution, making it ineffective for absorbing electromagnetic waves. Therefore, RGO-coated fabric restricts overall EMI shielding effectiveness (EMI SE), especially when absorption-dominated shielding is desired.

To address these deficiencies, a synergistic strategy involving the introduction of metallic nanoparticles and magnetic materials has been developed. Specifically, the incorporation of highly conductive silver nanoparticles (Ag) significantly enhances the electrical conductivity, thereby improving the dielectric loss and reflection attenuation [8,9]. Meanwhile, the integration of magnetic Fe_3_O_4_ nanoparticles introduces magnetic loss mechanisms, including natural resonance and eddy current loss, which facilitate the absorption of incident electromagnetic waves [10,11]. The combination of these three materials, RGO providing a conductive framework and dielectric loss, Ag NPs enhancing electrical conductivity, and Fe_3_O_4_ contributing magnetic loss, is expected to achieve a synergistic effect that optimizes both electrical conductivity and electromagnetic shielding performance. The resulting ternary coating features a rationally designed triple-layered architecture. The inner RGO layer, with its sheet-like structure, forms a conductive network directly on the fabric substrate; the intermediate Ag nanoparticle layer provides high electrical conductivity and contributes to dielectric loss; and the outer Fe_3_O_4_ layer introduces magnetic loss mechanisms. This hierarchical configuration enables complementary loss mechanisms combining conductive loss from RGO [12], dielectric loss from Ag, and magnetic loss from Fe_3_O_4_. The rationally designed triple-layer architecture (RGO inner, Ag intermediate, and Fe_3_O_4_ outer) creates a gradient impedance matching structure that minimizes surface reflection and maximizes internal absorption, thereby avoiding the secondary pollution problem inherent to reflection-dominated metal shields. The combination of these materials, with RGO providing a conductive framework and dielectric loss, Ag NPs enhancing electrical conductivity, and Fe_3_O_4_ contributing magnetic loss, is expected to achieve a synergistic effect that optimizes both electrical conductivity and electromagnetic shielding performance while shifting the shielding mechanism from reflection-dominated to absorption-dominated [13]. Consequently, the Fe_3_O_4_/Ag/RGO-coated fabric achieves substantially improved EMI shielding effectiveness, particularly in absorption-dominated shielding scenarios. Compared with existing EMI shielding materials, the Fe_3_O_4_/Ag/RGO-coated cotton fabric offers three key innovations: a triple-layer hierarchical structure enabling complementary loss mechanisms, absorption-dominated shielding (>99% absorption) that eliminates secondary electromagnetic pollution, and dopamine-assisted interfacial engineering ensuring strong adhesion without compromising flexibility. For wearable devices, it provides breathability and lightweight comfort; for military applications, its absorption dominance enables radar stealth and low observability, making it ideal for combat uniforms and tactical gear. However, investigations into the synergistic effects of ternary RGO/Ag/Fe_3_O_4_ composites coated onto fabric substrates via effective surface modification strategies remain limited. Additionally, achieving uniform and stable deposition of these nanomaterials onto cotton fabric remains a challenge due to the weak interfacial adhesion between the hydrophilic cotton fibers and the functional nanoparticles.

The preparation of nanoparticles onto fabrics is commonly achieved using conventional chemical reduction methods. However, traditional heating approaches such as water bath or oil bath suffer from drawbacks including slow heating rates, uneven temperature distribution, and substantial energy losses. In contrast, the microwave-assisted method offers several advantages, such as simplicity, rapid and uniform volumetric heating, fast reaction kinetics, short processing times, and high efficiency [14]. Therefore, a microwave-assisted approach was employed to load functional nanoparticles onto the fabric substrate, which is of great significance for achieving rapid, uniform, and energy-efficient deposition.

Recently, surface modification strategies have been developed to improve the binding affinity between nanomaterials and textile substrates. Among these, dopamine pretreatment has emerged as a particularly effective approach. Dopamine can self-polymerize under mild conditions to form a polydopamine (PDA) layer on the fabric surface, providing reactive functional groups that can strongly interact with functional nanomaterials [15]. This bio-inspired surface modification strategy significantly enables the stable and uniform coating of RGO, Ag, and Fe_3_O_4_ onto the cotton fabric substrate and enhances the interfacial adhesion between the cotton fabric and the functional nanoparticles, which is essential for the fabrication of high-performance composite materials. Nevertheless, there are few reports on integrating dopamine-pretreated cotton fabrics with Fe_3_O_4_/Ag/RGO nanocomposites for electrical conductivity and electromagnetic shielding performance.

In this study, Fe_3_O_4_/Ag/RGO nanocomposites with a triple-layer structure were coated on cotton fabrics. In detail, the cotton fabric was pretreated with dopamine to introduce active functional groups onto the fiber surface to enhance interfacial adhesion. Subsequently, reduced graphene oxide (RGO) was coated onto the dopamine-treated cotton fabric via a microwave-assisted method. Next, silver nanoparticles were deposited onto the GO-coated cotton fabric using a microwave-assisted chemical reduction technique to form Ag/RGO-coated fabric. Finally, Fe_3_O_4_ nanoparticles were deposited onto the as-prepared Ag/RGO-coated fabric via a coprecipitation method, and a tenary Fe_3_O_4_/Ag/RGO nanocomposite-coated fabric was obtained. The electrical conductivity and EMI shielding properties of the ternary Fe_3_O_4_/Ag/RGO nanocomposite-coated fabrics were evaluated. The results demonstrate that the synergistic combination of RGO, Ag, and Fe_3_O_4_ leads to superior electromagnetic shielding effectiveness exceeding 45 dB across the 1–18 GHz frequency range, while also achieving excellent electrical conductivity. This work provides a promising strategy for the development of high-performance, flexible, and lightweight EMI shielding materials suitable for next-generation wearable electronics and advanced protective applications.

## 2. Experimental

### 2.1. Chemicals and Materials

The plain weave cotton fabric was purchased from Hangzhou Xuri Textile Co., Ltd. (Hangzhou, China). The chemicals, including FeCl_3_·6H_2_O, NH_3_·H_2_O, FeCl_2_, ethanol, acetone, silver nitrate, trisodium citrate dihydrate, and dopamine, were all purchased from Chengdu Kelong Chemical Reagent Company (Chengdu, China). Graphene oxide was purchased from Suzhou Tanfeng Graphene Technology Co., Ltd. (Suzhou, China). All the chemical reagents were of analytical grade.

### 2.2. Pretreatment of Cotton Fabric

The cotton fabric was cut into pieces of 5 cm × 5 cm and ultrasonically cleaned in a mixed solution of ethanol and acetone (1:1 by volume) for 30 min. The fabric was then washed with deionized water and dried for use. A 3 g/L dopamine aqueous solution was prepared, and the pH was adjusted to 8.5. The cleaned cotton fabric was immersed in the dopamine solution at room temperature for 24 h and then washed with deionized water, dried, and used. The obtained fabric is named as pretreated cotton fabric.

### 2.3. Coating of Reduced Graphene Oxide on Fabric

A total of 0.05 g of graphene oxide was dissolved in 100 g of deionized water and ultrasonically dispersed for 30 min. Subsequently, 0.05 g of sodium citrate was added, and the pretreated cotton fabric was immersed in the mixed solution with thorough stirring. The reaction solution was placed in an 800 W microwave oven and reacted for 10 min. To ensure uniform coating and prevent violent boiling, heating was paused every 2 min to turn over the fabric surface. The reduced graphene oxide (RGO)-coated cotton fabric was formed. Finally, the RGO-coated cotton fabric was dried at 60 °C.

### 2.4. Deposition of Silver Nanoparticles on RGO-Coated Fabric

AgNO_3_ solutions with concentrations of 6 g/L were prepared, respectively, and trisodium citrate was added at a ratio of 1:2 (AgNO_3_: trisodium citrate). Sodium citrate was added in excess (AgNO_3_:sodium citrate = 1:2) to reduce GO to RGO and subsequently reduce Ag^+^ to Ag^0^. Although microwave irradiation alone can reduce Ag^+^ without a reducing agent, sodium citrate ensured uniform nucleation and prevented nanoparticle aggregation. The RGO-coated cotton fabric was immersed in the above mixed solution with thorough stirring. The reaction solution was placed in an 800 W microwave oven and reacted for 10 min. To ensure uniform coating and prevent violent boiling, heating was paused every 2 min to turn over the fabric surface. Subsequently, silver (Ag) nanoparticles were deposited on RGO-coated fabric, which was named Ag/RGO-coated fabric. Finally, the Ag/RGO-coated fabric was dried at 60 °C.

### 2.5. Synthesis of Fe_3_O_4_/Ag/RGO Nanocomposite-Coated Cotton Fabric

FeCl_3_ and FeCl_2_ were dissolved in distilled water at a molar ratio of 1.85:1 under nitrogen protection. The solution was heated to 60 °C in a constant temperature water bath. NH_3_·H_2_O was slowly added dropwise until the pH reached 11 under rapid stirring. The temperature was rapidly increased to 85 °C under stirring or aging for 1 h. The resulting mixture was washed with ethanol and separated using a magnet and was then sealed and stored. The Ag/RGO-coated fabric was immersed in the Fe_3_O_4_ nanoparticles and dispersed in ethanol solution for 12 h. Fe_3_O_4_ was then coated onto the Ag/RGO-coated fabric surface using a spraying method and dried in a vacuum oven at 60 °C; thus, Fe_3_O_4_/Ag/RGO-coated cotton fabric was formed.

To enable a comparative evaluation of electrical conductivity and electromagnetic shielding performance, cotton fabrics coated with a single layer of RGO, Ag, and Fe_3_O_4_ were used using similar procedures. In addition, triple types of bilayer coating, designated as Ag/RGO, Fe_3_O_4_/RGO, and Ag/Fe_3_O_4_, were coated on cotton fabric, as well as a triple-layer Ag/RGO/Fe_3_O_4_-coated fabric. In detail, Fe_3_O_4_/RGO-coated fabric was prepared by immersing RGO-coated fabric in Fe_3_O_4_/ethanol dispersion (from the same coprecipitation method) for 12 h, followed by drying at 60 °C. Ag/Fe_3_O_4_-coated fabric was prepared by immersing Fe_3_O_4_-coated fabric in AgNO_3_/trisodium citrate solution (6 g/L AgNO_3_, AgNO_3_:citrate = 1:2 weight ratio) and treating it in an 800 W microwave oven for 10 min, turning the fabric every 2 min to ensure uniform coating and then drying at 60 °C.

### 2.6. Characterization

The surface morphologies of the RGO-coated cotton fabric, RGO/Ag-coated cotton fabric, Ag/Fe_3_O_4_-coated cotton fabric, and Fe_3_O_4_/Ag/RGO-coated cotton fabric were observed using a JSM-5900LV scanning electron microscope (SEM, JEOL, Tokyo, Japan). The crystal structure of the Fe_3_O_4_/Ag/RGO-coated cotton fabric was characterized using an X’Pert Pro MPD X-ray diffractometer (XRD, Philips, Amsterdam, The Netherlands). The measurement was performed with Cu Kα radiation at a tube voltage of 40 kV, a tube current of 30 mA, and a scanning speed of 10°/min. The chemical composition of the Fe_3_O_4_/Ag/RGO-coated cotton fabric was analyzed using an EDX analyzer (SEM attachment, JSM-5900LV, JEOL, Tokyo, Japan). The thermal stabilities of the bilayer coating (Fe_3_O_4_/RGO, Ag/RGO, and Fe_3_O_4_/Ag) cotton fabrics and the triple-layer coating (Ag/RGO/Fe_3_O_4_ and Fe_3_O_4_/Ag/RGO) fabrics were analyzed using a DTG-60(H) differential thermal-thermogravimetric analyzer over a temperature range from room temperature to 450 °C at a heating rate of 10 °C/min.

### 2.7. Electrical Resistance and EMI Shielding Effectiveness Properties Tests

The electrical resistances of the dopamine-pretreated cotton fabric, single-layer coating (RGO, Ag, and Fe_3_O_4_) cotton fabrics, bilayer coating (Fe_3_O_4_/RGO, Ag/RGO, and Fe_3_O_4_/Ag) cotton fabrics, and triple-layer coating (Ag/RGO/Fe_3_O_4_ and Fe_3_O_4_/Ag/RGO) fabrics were measured using an RTS-9 dual-configuration four-probe resistivity tester (Guangzhou Four-Probe Technology Co., Ltd., Guangzhou, China). The EMI shielding properties of the single-layer coating (RGO, Ag, and Fe_3_O_4_) cotton fabrics, bilayer coating (Fe_3_O_4_/RGO, Ag/RGO, and Fe_3_O_4_/Ag) cotton fabrics, and triple-layer coating (Ag/RGO/Fe_3_O_4_ and Fe_3_O_4_/Ag/RGO) fabrics were measured using a vector network analyzer (Agilent E5063A ENA, Santa Clara, CA, USA). To evaluate whether the shielding mechanism is dominated by absorption or reflection, the absorption and reflection values of electromagnetic waves were determined for the coated fabrics with excellent electromagnetic shielding effectiveness.

## 3. Results and Discussion

### 3.1. Formation Process of Fe_3_O_4_/Ag/RGO-Coated Cotton Fabrics

**Figure 1:**
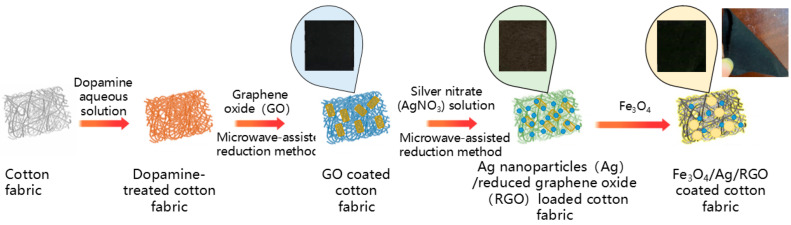
Formation processes of Fe_3_O_4_/Ag/RGO-coated cotton fabric.

The formation process of the Fe_3_O_4_/Ag/RGO-coated cotton fabric involves a sequential multi-step assembly strategy, as illustrated in Figure 1. Each step is designed to introduce specific functional components onto the fabric surface, with distinct formation mechanisms underlying each deposition process. The pristine cotton fabric is first immersed in a dopamine solution under weak alkaline conditions (pH ≈ 8.5). Dopamine undergoes self-polymerization to form a polydopamine (PDA) layer that uniformly coats the cotton fibers. The PDA layer serves as a bio-inspired adhesive agent, providing abundant catechol and amine functional groups that act as anchoring sites for subsequent nanoparticle deposition. The formation mechanism involves oxidative polymerization of dopamine, with the PDA layer adhering strongly to the cotton fibers through hydrogen bonding and covalent interactions.

After that, graphene oxide (GO) is coated onto the PDA-coated cotton fabric using a microwave-assisted reduction method, with sodium citrate employed as a reducing agent. GO is simultaneously reduced to reduced graphene oxide (RGO) [16], which is deposited on black cotton fabric during this process. The microwave irradiation provides rapid and uniform volumetric heating, accelerating the reduction kinetics and promoting the formation of a conductive RGO network on the fabric surface. The reduction mechanism involves the donation of electrons from citrate ions to GO, eliminating oxygen-containing functional groups and restoring the conjugated sp^2^ carbon structure. The PDA layer enhances RGO adhesion through π-π interactions and hydrogen bonding between the catechol groups and the RGO nanosheets.

Subsequently, silver nanoparticles (Ag NPs) are deposited onto the RGO-coated fabric using a microwave-assisted chemical reduction method. Silver ions (Ag^+^) are first adsorbed onto the negatively charged RGO and PDA surfaces via electrostatic interactions [16]. Ag^+^ ions bind to three types of sites on the RGO/PDA-coated fabric, negatively charged oxygen groups (-COO^−^, -O^−^) on RGO via electrostatic attraction, and amine/catechol groups on PDA via chelation. Sodium citrate served as both a reducing agent and a stabilizing agent. The PDA and RGO layers provide anchoring sites that prevent nanoparticle aggregation. Rapid and uniform heating facilitates the reduction in Ag^+^ to Ag^0^ under microwave irradiation, leading to the nucleation and growth of spherical Ag nanoparticles uniformly dispersed on the fabric surface. The Ag/RGO-coated cotton fabric exhibits a gray-black appearance due to the presence of Ag in the outer layer and RGO in the inner layer, respectively.

Finally, Fe_3_O_4_ nanoparticles are synthesized via a coprecipitation method. Typically, Fe^2+^ and Fe^3+^ salts are mixed in an alkaline solution, leading to the formation of Fe_3_O_4_ nanoparticles through the coprecipitation reaction. The as-prepared Fe_3_O_4_ nanoparticles are then deposited onto the pre-coated Ag/RGO fabric through a spray coating process, which involves immersing the fabric into a suspension of Fe_3_O_4_ nanoparticles followed by drying. The triple-layer Fe_3_O_4_/Ag/RGO nanocomposite-coated cotton fabric displays a black color with Fe_3_O_4_ as the outermost layer. The adhesion of Fe_3_O_4_ nanoparticles with a high loading amount to the fabric surface is primarily driven by intermolecular electrostatic interactions and hydrogen bonding between the Fe_3_O_4_ nanoparticles and the underlying Ag/RGO layers. The relatively high loading amount of Fe_3_O_4_ can be attributed to the abundance of binding sites provided by the previously deposited layers and the favorable electrostatic interactions between the positively charged Fe_3_O_4_ surface (under acidic conditions) and the negatively charged underlying components. Fe_3_O_4_/Ag/RGO-coated cotton fabric can be attracted by a permanent magnet, indicating that Fe_3_O_4_ is successfully coated onto the cotton fabric as the outer layer, endowing the fabric with the paramagnetic properties of Fe_3_O_4_.

The resulting ternary coating features a hierarchical architecture: the innermost PDA layer ensures strong adhesion to the cotton substrate; the intermediate RGO layer forms a conductive network; the Ag nanoparticle layer provides high electrical conductivity and contributes to dielectric loss; and the outermost Fe_3_O_4_ layer introduces magnetic loss capability. This rationally designed multi-layer configuration enables complementary loss mechanisms and enhanced impedance matching, contributing to superior electromagnetic shielding performance.

In addition, the microwave-assisted method used offers significant advantages for scalable production, including rapid volumetric heating (10 min reaction time), energy efficiency, and uniform nanoparticle deposition. This approach is compatible with continuous roll-to-roll processing, making it suitable for industrial-scale manufacturing of functional textiles.

### 3.2. Surface Morphology

**Figure 2:**
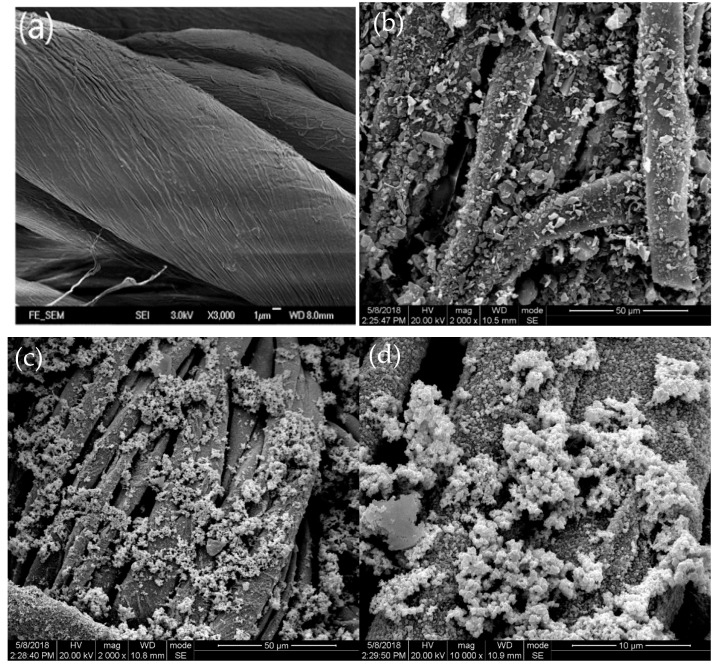

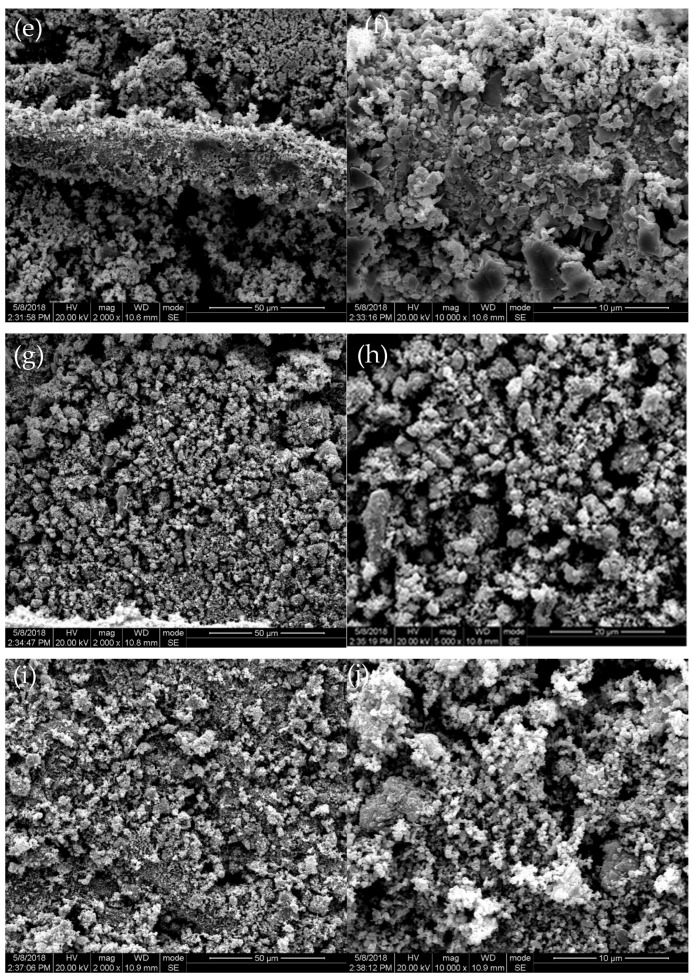
(**a**) SEM images of original cotton fibers at magnifications of 5000, (**b**) RGO-coated cotton fabric at magnifications of 10,000, Ag-coated cotton fabric without RGO at magnifications of (**c**) 5000 and (**d**) 10,000, Ag/RGO-coated cotton fabric at magnification of (**e**) 2000 and (**f**) 10,000, Fe_3_O_4_/Ag-coated cotton fabric without RGO at magnification of (**g**) 2000 and (**h**) 10,000, and Fe_3_O_4_/Ag/RGO-coated cotton fabric at magnification of (**i**) 2000 and (**j**) 10,000.

Figure 2 shows the SEM images of cotton fibers, single-layer coating (RGO, Ag, and Fe_3_O_4_) cotton fabrics, bilayer coating (Fe_3_O_4_/RGO, Ag/RGO, and Fe_3_O_4_/Ag) cotton fabrics, and triple-layer coating (Ag/RGO/Fe_3_O_4_ and Fe_3_O_4_/Ag/RGO) fabrics. Figure 2a presents the surface morphology of pristine cotton fibers, revealing that the cotton fibers are not cylindrical but exhibit a twisted helical structure with natural folds along the fiber length, which physically facilitates the mechanical interlocking of nanoparticles. A polydopamine polymer film is formed wrapping around the fiber surface after dopamine pretreatment, and the adhesive nature of this film facilitates deposition. The PDA layer not only smoothens the fiber surface but also introduces catechol and amine functional groups that act as chemical anchoring sites. A substantial amount of reduced graphene oxide in nanosheet-like forms is uniformly deposited on the cotton fibers, making the fiber surface rough, as shown in Figure 2b. Additionally, RGO possesses a negatively charged structure and disperses easily in aqueous solution, resulting in relatively uniform loading on the fabric surface. Furthermore, the negative charge can interact with the functional groups of the fabric, thereby enhancing the immobilization of RGO. RGO nanosheets form a dense and continuous coating on the PDA-modified cotton fiber surface (Figure 2b), with overlapping sheets covering most of the fiber. The uniform and dense coverage of RGO nanosheets on PDA-coated fibers (Figure 2b) is attributed to the strong π-π stacking and hydrogen bonding between RGO and PDA, as RGO sheets tend to align parallel to the fiber axis to maximize contact area. However, some microscale-exposed PDA areas remain due to the wrinkled morphology of RGO, which actually serve as additional anchoring sites for subsequent Ag nanoparticle deposition.

As observed in Figure 2c,d, silver nanoparticles are spherical in shape and deposited on the cotton fabric surface without RGO modification, leading to uneven loading on the fabric surface. Ag nanoparticles deposited directly onto cotton fibers without RGO modification (Figure 2c,d) show significant aggregation and uneven distribution, indicating weak interfacial adhesion between Ag and pristine cellulose. The particle size is approximately 100 nm. Figure 2e,f show that silver nanoparticles are successfully coated on the RGO-coated fabric surface. Compared with the Ag-coated fabric, the Ag nanoparticles coated on the RGO-modified cotton fiber surface are uniform and dense. This phenomenon can be explained by the fact that RGO-modified fibers with a negative charge serve as an excellent substrate for Ag ions with a positive charge, which can easily attach to the RGO surface through strong electrostatic forces. In addition, when deposited onto RGO-coated fibers (Figure 2e,f), Ag nanoparticles become spherical, uniform, and densely packed, suggesting that the RGO layer not only provides electrostatic attraction for Ag^+^ but also serves as a nucleation template that prevents nanoparticle aggregation.

Both silver nanoparticles and Fe_3_O_4_ nanoparticles with spherical shapes are uniformly and densely deposited on the cotton fabric surface without RGO coating, as shown in Figure 2g,h. The particle size of Fe_3_O_4_ is approximately 200 nm. The relatively higher loading amount of Fe_3_O_4_ nanoparticles may be attributed to the strong chelation between the polydopamine (PDA) layer and Fe^3+^/Fe^2+^ ions during the coating. The catechol and amine groups in PDA serve as efficient anchoring sites for iron ions, promoting the growth of Fe_3_O_4_ nanoparticles with high density and strong interfacial adhesion. Figure 2i,j reveal that silver nanoparticles and Fe_3_O_4_ nanoparticles are uniformly coating on the RGO-coated fabric surface, indicating that the Fe_3_O_4_/Ag/RGO triple-layer composite material was successfully coated onto the cotton fabric. The Fe_3_O_4_ nanoparticles deposited on Ag/RGO-coated fibers (Figure 2i,j) exhibit a relatively larger particle size (~200 nm) compared with Ag nanoparticles (~100 nm), yet they remain uniformly distributed without severe agglomeration. This indicates that the underlying Ag/RGO/PDA layers offer sufficient anchoring sites, primarily through hydrogen bonding and coordination interactions, to stabilize the Fe_3_O_4_ nanoparticles.

### 3.3. Crystal Structure

**Figure 3:**
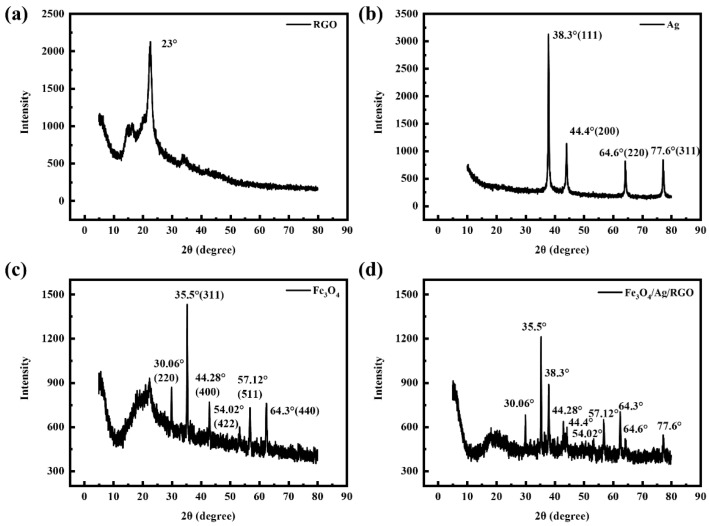
XRD patterns of (**a**) RGO-, (**b**) Ag-, (**c**) Fe_3_O_4_-, and (**d**) Fe_3_O_4_/Ag/RGO-coated cotton fabrics.

Figure 3a shows the XRD pattern of the RGO-coated cotton fabric characterized by X-ray diffraction. Reduced graphene oxide exhibits different characteristic peaks depending on the reduced functional groups, typically around 23°. As can be seen in the figure, a sharp peak appears at approximately 2θ = 23° [16], indicating that RGO is successfully coated onto the cotton fabric. In addition, the broad diffraction peak of RGO appears at approximately 2θ = 23°, which is slightly higher than the theoretical value for ideal graphene (2θ ≈ 20–22°). This upshift suggests a reduced interlayer spacing of the RGO nanosheets, likely due to strong π-π stacking interactions between RGO and the underlying polydopamine (PDA) layer. The PDA anchors RGO through its aromatic rings, pulling the nanosheets closer to the fiber surface and compressing the interlayer distance. The characteristic peak of cellulose at 22° overlaps with the characteristic peak of RGO at approximately 23°. The additional peaks are attributed to the cellulose of the cotton fabric.

Figure 3b presents the XRD pattern of the Ag-coated cotton fabric. The XRD pattern reveals four characteristic diffraction peaks of Ag at 2θ values of 38.30°, 44.40°, 64.60°, and 77.60°. Compared with the standard pattern of elemental silver (JCPDS Card No. 04-0783), these peaks correspond to the (111), (200), (220), and (311) crystal planes of face-centered cubic metallic silver [17], respectively. This indicates that silver nanoparticles with a face-centered cubic structure are successfully prepared and coated onto the cotton fabric. Additionally, the characteristic Ag diffraction peaks (111), (200), (220), and (311) are sharp and well-defined, indicating high crystallinity of the Ag nanoparticles. Notably, no peak shifting or broadening associated with Ag oxidation is observed, confirming that the Ag nanoparticles remain chemically stable. This stability is attributed to the protective effect of the PDA and RGO layers, which not only anchor Ag nanoparticles through electrostatic and chelation interactions but also prevent direct contact between Ag and the alkaline Fe_3_O_4_ precursor solution during subsequent deposition.

The XRD pattern of the Fe_3_O_4_-coated cotton fabric is shown in Figure 3c. The XRD pattern reveals six distinct characteristic diffraction peaks at 2θ values of 30.06°, 35.50°, 44.28°, 54.02°, 57.12°, and 64.30°, all of which correspond to the characteristic peaks of Fe_3_O_4_ crystals. The corresponding crystal plane indices are (220), (311), (400), (422), (511), and (440), respectively, indicating a face-centered cubic structure [18]. These results are consistent with the standard diffraction card for Fe_3_O_4_ crystals (JCPDS No. 75-1610), demonstrating that Fe_3_O_4_ is successfully coated onto the fabric. The relatively broad peak widths suggest nanocrystalline Fe_3_O_4_ with small grain sizes, which is favorable for magnetic loss mechanisms. Importantly, no additional peaks corresponding to Fe_2_O_3_ or other iron oxides are detected, indicating that the Fe_3_O_4_ nanoparticles remain phase-pure.

Figure 3d shows the XRD pattern of the Fe_3_O_4_/Ag/RGO-coated cotton fabric. It can be observed that the fabric exhibits characteristic peaks of Fe_3_O_4_ at 2θ values of 30.06°, 35.50°, 44.28°, 54.02°, 57.12°, and 64.30° and characteristic peaks of Ag at 38.30°, 44.40°, 64.60°, and 77.60° [13]. A broad peak corresponding to cellulose appears around 20°. The characteristic peak of RGO is not clearly visible. The phenomenon can be explained by the fact that RGO is located as the innermost layer, covered by dense Ag and Fe_3_O_4_ coatings; the strong π-π stacking interaction between RGO and PDA reduces the electron density contrast; and the characteristic peak of RGO partially overlaps with the broad cellulose peak of the cotton substrate. This observation indirectly supports the hierarchical interfacial structure, where each layer is tightly bound to the adjacent layer through complementary interfacial interactions (π-π stacking, hydrogen bonding, electrostatic attraction, and coordination) rather than existing as physically mixed or loosely attached components.

### 3.4. Chemical Composition

**Figure 4:**
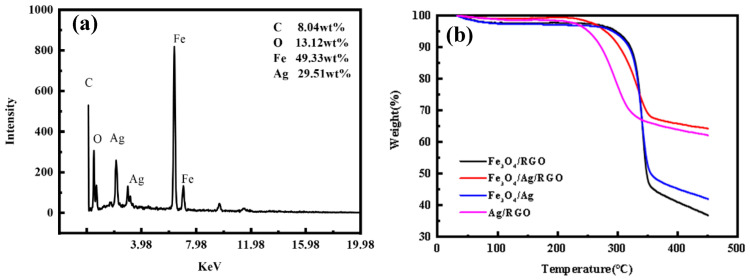
(**a**) EDX spectrum of Fe_3_O_4_/Ag/RGO-coated cotton fabric, (**b**) TGA curves of RGO/Ag-, Ag/Fe_3_O_4_-, RGO/Fe_3_O_4_-, and RGO/Ag/Fe_3_O_4_-coated cotton fabrics.

Figure 4a shows the EDX spectrum of the Fe_3_O_4_/Ag/RGO-coated cotton fabric. It can be observed that the coating on the cotton fabric contains four elements: C, O, Fe, and Ag. Among them, Fe and Ag originate from Fe_3_O_4_ and silver nanoparticles, respectively, C mainly comes from RGO and the cotton fabric, and O mainly comes from RGO, Fe_3_O_4_, and the cotton fabric. Based on the weight ratio of O and Fe elements, the magnetic component can be identified as Fe_3_O_4_, indicating that RGO, Fe_3_O_4_, and Ag are all successfully coated onto the cotton fabric. Based on EDX analysis (Figure 4a), the mass ratio of Ag to Fe_3_O_4_ on the RGO surface is approximately 1:2.3, corresponding to Ag 29.5 wt% and Fe_3_O_4_ 68.2 wt% of the total coating mass. In terms of mass fraction, Fe_3_O_4_ accounts for a relatively large proportion, which may be due to its higher specific gravity and the fact that it forms a dense coating on the surface as the outermost layer material. Since RGO is located in the innermost layer of the composite with dense Ag and Fe_3_O_4_ coatings on it, the detected carbon content is relatively low. The relatively low carbon content detected from the surface (despite RGO being the innermost layer) suggests that the outer Fe_3_O_4_ and Ag layers are sufficiently dense and continuous, limiting X-ray penetration. This further confirms the strong interfacial adhesion between layers, as no delamination or cracking that would expose the underlying RGO layer is observed. Based on the weight ratio of the O and Fe elements, it can be inferred that there are other sources of oxygen in addition to Fe_3_O_4_, which may originate from oxygen-containing functional groups in incompletely reduced graphene oxide, as well as CO_2_ and O_2_ from the air.

### 3.5. Thermogravimetric Analysis

Figure 4b presents the thermogravimetric analysis curves of the Ag/RGO-coated cotton fabric, Fe_3_O_4_/Ag-coated cotton fabric, Fe_3_O_4_/RGO-coated cotton fabric, and Fe_3_O_4_/Ag/RGO-coated cotton fabric. The TGA curves reveal three distinct thermal degradation stages for all coated fabrics. A slight mass loss of approximately 0.2–1.5% is observed due to evaporation of absorbed moisture. In Stage II (300–400 °C), a sharp and rapid mass loss of 40–50% occurs, corresponding to the thermal decomposition of cotton cellulose fibers. In Stage III (above 400 °C), the curves reach a plateau, where the remaining mass represents the inorganic coating residues (RGO, Ag, and Fe_3_O_4_). The Fe_3_O_4_/Ag/RGO triple-layer coating exhibits the highest residual mass (64.21% at 450 °C), indicating the greatest total coating mass. This is attributed to the synergistic effect of the PDA anchor layer, which provides abundant binding sites for all three components (RGO via π-π stacking, Ag via electrostatic/chelation, and Fe_3_O_4_ via hydrogen bonding/coordination). The Ag/RGO coating shows a residual mass of 62.07% due to the dense and uniform Ag network formed on the RGO surface. In contrast, the Fe_3_O_4_/Ag (41.90%) and Fe_3_O_4_/RGO (36.7%) coatings exhibit lower residuals, because without the RGO layer (for Ag/Fe_3_O_4_) or without the Ag layer (for Fe_3_O_4_/RGO), the deposition efficiency and interfacial adhesion are reduced. The thermal stability of all coatings exceeds 400 °C, which is sufficient for most wearable and military applications where fabrics may be exposed to elevated temperatures during use.

### 3.6. Electrical Resistance

**Figure 5:**
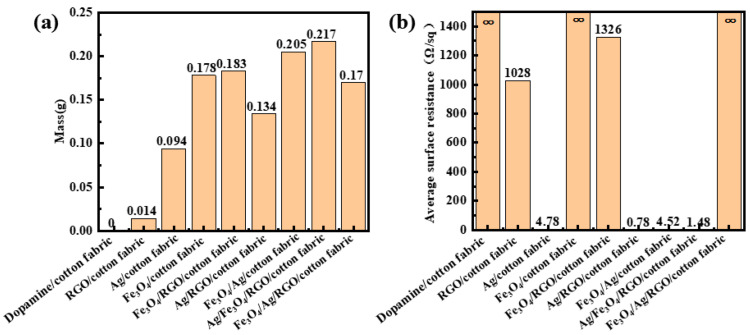
(**a**) Deposit weight of different nanomaterial-coated cotton fabrics. (**b**) Average surface resistance of different nanomaterial-coated cotton fabrics.

Figure 5 shows the coating amounts and surface resistance values of the dopamine-pretreated cotton fabric, single-layer coating (RGO, Ag, and Fe_3_O_4_) cotton fabrics, bilayer coating (Fe_3_O_4_/RGO, Ag/RGO, and Fe_3_O_4_/Ag) cotton fabrics, and triple-layer coating (Ag/RGO/Fe_3_O_4_ and Fe_3_O_4_/Ag/RGO) fabrics. As can be seen in Figure 5, among the single-layer material coated cotton fabrics, RGO exhibits extremely weak electrical conductivity, and its surface resistance (1028 Ω/sq) is relatively high. It is known that ideal graphene has a planar hexagonal structure, where sp^2^ hybridized carbon atoms form σ bonds, and the electrons in the remaining p orbitals form a large π bond. The freely mobile π electrons endow graphene with excellent electrical conductivity. However, it is difficult to obtain an ideal two-dimensional graphene structure during the fabric loading, and some oxygen-containing functional groups remain unreduced during the reduction. Additionally, RGO nanoparticles tend to aggregate in solution, preventing RGO from establishing a conductive network on the cotton fabric surface, resulting in low coating content (0.014 g), which is poor electrical conductivity. For Fe_3_O_4_-coated fabric, although the content of Fe_3_O_4_ coating is not low (0.178 g), surface resistance is very high (∞), and Fe_3_O_4_-coated fabric is almost not electrically conductive. The phenomenon can be explained by the fact that Fe_3_O_4_ is an ionic crystal with a face-centered cubic structure. In the solid state, the ions at the lattice nodes can only vibrate and lack freely mobile electrons; thus, they cannot conduct electricity. The silver nanoparticle-coated cotton fabric exhibits excellent electrical conductivity with a low sheet resistance of 4.78 Ω/sq. This is attributed to the high deposition content of Ag nanoparticles (0.094 g) and the formation of a dense and continuous conductive network on the fiber surface, which provides efficient pathways for electron transport.

Among the double-layer composites, although the contents of Fe_3_O_4_/RGO coating and Fe_3_O_4_/Ag are relatively high (0.183 g and 0.205 g), Fe_3_O_4_/RGO-coated cotton fabric and Fe_3_O_4_/Ag-coated cotton fabric show no significant change in electrical resistance compared with the single-layer RGO-coated and Ag-coated cotton fabrics, with even a slight decrease in electrical conductivity. This is because the presence of Fe_3_O_4_ does not provide additional freely mobile electrons and even somewhat hinders the movement of existing free electrons, resulting in a slight increase in electrical resistance.

The surface resistance of Ag/RGO-coated cotton fabrics reaches 0.78 Ω/sq, representing a significant improvement in conductivity after the combination of Ag and RGO. This may be attributed to the reason that the RGO nanosheets coated on the cotton fabric provide abundant functional groups (e.g., hydroxyl, carboxyl) that serve as nucleation sites, promoting the uniform and dense deposition of silver nanoparticles. This results in a high coating content of 0.134 g. In addition, a synergistic effect arises from the point–plane contact configuration between Ag nanoparticles and RGO nanosheets, which not only increases the density of conductive pathways but also reduces the contact resistance among adjacent conductive fillers. Consequently, a more interconnected and continuous conductive network is formed across the fabric surface, enabling efficient charge carrier transport and leading to the substantially enhanced electrical conductivity.

Among the triple-layer composite-coated cotton fabrics, the electrical resistance of Ag/Fe_3_O_4_/RGO-coated fabric (namely, the fabric coated first with RGO, then with Fe_3_O_4_, and finally with Ag nanoparticles) is relatively high, which is not electrically conductive. It was observed that the solution of Ag ions and sodium citrate turns yellow when loading Ag nanoparticles using the microwave method after Fe_3_O_4_ loading. This may be attributed to the oxidation of Fe_3_O_4_ to Fe^3+^ in the aqueous solution under microwave heating, which also prevents the reduced silver nanoparticles from being coated onto the fabric. Therefore, the deposit content of Ag/Fe_3_O_4_/RGO is lower than that of Fe_3_O_4_/RGO.

However, for Fe_3_O_4_/Ag/RGO-coated fabric, Fe_3_O_4_ can be successfully deposited on Ag/RGO-coated cotton fabric due to favorable electrostatic interactions between the positively charged Fe_3_O_4_ surface and the negatively charged underlying Ag/RGO. Although the deposit amount of Fe_3_O_4_/Ag/RGO is higher than that of Ag/RGO, the electrical resistance of the Fe_3_O_4_/Ag/RGO-coated fabric is higher than that of the Ag/RGO-coated fabric. This phenomenon can be explained by the fact that Fe_3_O_4_ is deposited as the outermost layer, and its intrinsic electrical conductivity is substantially lower than that of Ag and RGO, which thus shields the underlying highly conductive Ag/RGO network, forcing charge carriers to traverse the less conductive Fe_3_O_4_ layer. Additionally, the incorporation of Fe_3_O_4_ disrupts the continuous conductive network originally formed by Ag and RGO. The Fe_3_O_4_ nanoparticles act as physical barriers, reducing the effective contact between Ag nanoparticles and RGO nanosheets. Furthermore, the favorable point–plane contact configuration between Ag and RGO, which is key to the low resistance of Ag/RGO, is compromised, as Fe_3_O_4_ nanoparticles interpose between or encapsulate the conductive fillers. Consequently, the overall contact resistance and interfacial energy barrier increase, leading to a higher resistance despite the greater total deposit content.

### 3.7. Electromagnetic Interference Shielding Effectiveness

Electromagnetic shielding generally employs low-resistance conductive materials to reflect and guide the flow of electromagnetic energy, reducing electromagnetic radiation generated by a radiation source from one region to another to mitigate radiation hazards [7]. It is typically characterized by shielding effectiveness (SE), expressed as SE (dB) = R + A + B, where R represents the reflection loss of electromagnetic wave energy by the material. A represents the absorption loss of electromagnetic wave energy, and B represents the transmission loss of electromagnetic waves within the shielding material (when A > 10 dB, B can be neglected) [2,10]. Based on shielding principles, there are two main shielding mechanisms for current electromagnetic shielding materials: one is reflection at the incident surface to achieve electromagnetic shielding; the other is absorption of electromagnetic waves within the shielding material to achieve shielding effectiveness. It is generally considered that there is essentially no electromagnetic shielding effect when SE is less than 10 dB; SE below 30 dB corresponds to low electromagnetic shielding materials, and an effective shielding effect requires an SE value of at least 35 dB [15]. Good electromagnetic shielding materials should possess high electrical conductivity and high magnetic permeability.

**Figure 6:**
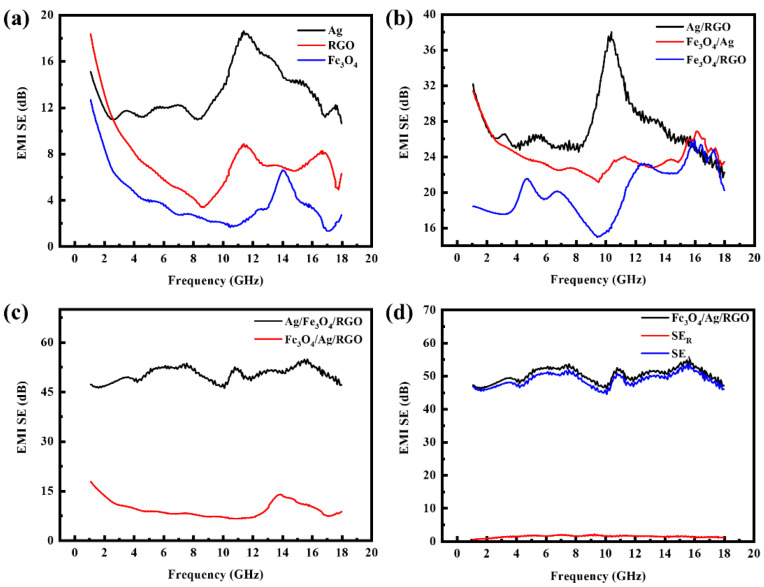
(**a**) EMI shielding effectiveness curves of single-layer (RGO, Ag, and Fe_3_O_4_)-coated cotton fabrics, (**b**) EMI shielding effectiveness curves of double-layer (RGO/Ag, Ag/Fe_3_O_4_, and RGO/Fe_3_O_4_) nanocomposite-coated cotton fabrics, (**c**) EMI shielding effectiveness curves of triple-layer (Ag/Fe_3_O_4_/RGO and Fe_3_O_4_/Ag/RGO) nanocomposite-coated fabrics, (**d**) spectral absorption (SE_A_) and reflection (SE_R_) of electromagnetic waves by Fe_3_O_4_/Ag/RGO-coated cotton fabrics.

Figure 6a shows the electromagnetic shielding performance of single-layer material-coated cotton fabrics. The Fe_3_O_4_-coated and RGO-coated cotton fabrics exhibit SE values below 10 dB across the 1–18 GHz range and cannot be directly used as electromagnetic shielding materials. The phenomenon can be explained by the fact that the Fe_3_O_4_-coated cotton fabric alone possesses neither high electrical conductivity nor high magnetic permeability, resulting in poor electromagnetic shielding performance. Similarly, the RGO-coated cotton fabric exhibits limited shielding effectiveness against electromagnetic waves due to poor electrical conductivity, which cannot generate induced currents. As can be seen in the figure, the Ag-coated fabric already exhibits a certain electromagnetic shielding capability, with its SE values exceeding 10 dB across the 1–18 GHz frequency range and reaching a maximum of 19 dB at 12 GHz. This is attributed to the high electrical conductivity of Ag, which generates significant induced currents under the action of electromagnetic waves. According to Lenz’s law, these currents weaken the penetration of electromagnetic waves.

Figure 6b shows the EMI shielding effectiveness curves of the double-layer composite-coated cotton fabrics. It can be observed that the double-layer composites exhibit good electromagnetic shielding performance. The Fe_3_O_4_/RGO-coated cotton fabric exhibits SE values greater than 14 dB across the 1–18 GHz range, reaching a maximum of 25 dB at 16 GHz. The Fe_3_O_4_/Ag-coated cotton fabric exhibits EMI SE values greater than 20 dB across the 1–18 GHz range, reaching a maximum of 26 dB at 16 GHz. This is because the Fe_3_O_4_/Ag-coated cotton fabric exhibits increased magnetic permeability after loading with Fe_3_O_4_ compared with the Ag-coated fabric, thereby improving its electromagnetic shielding effectiveness. However, the Ag/RGO-coated cotton fabric exhibits EMI SE values greater than 24 dB within the 1–18 GHz range, reaching a maximum of 38 dB at the frequency of 10 GHz. This may be attributed to the increased electrical conductivity of the RGO/Ag-coated cotton fabric, which enhances its electromagnetic shielding performance.

Figure 6c shows the electromagnetic shielding curves of the triple-layer composite-coated cotton fabrics. It can be observed that the Ag/Fe_3_O_4_/RGO-coated cotton fabric exhibits almost no electromagnetic shielding effectiveness due to the oxidation of Fe_3_O_4_ during the reduction in Ag ions, which prevents the successful loading of both Fe_3_O_4_ and silver nanoparticles. However, the Fe_3_O_4_/Ag/RGO-coated cotton fabric exhibits excellent electromagnetic shielding performance, with electromagnetic shielding effectiveness exceeding 45 dB across the entire 1–18 GHz frequency range and reaching 55 dB at 16 GHz, demonstrating effective electromagnetic shielding capability. This is explained by the fact that both electrical conductivity and magnetic permeability are significantly enhanced after the combination of these triple materials, and the resulting synergistic effect greatly improves the electromagnetic shielding performance, meeting the requirements for electromagnetic shielding materials with broad frequency bandwidth and wide applicability.

Figure 6d shows the electromagnetic wave absorption and reflection diagrams of the Fe_3_O_4_/Ag/RGO-coated cotton fabric. Here, SE_R_ represents the reflection loss of electromagnetic waves by the material, and SE_A_ represents the absorption loss of electromagnetic waves. As can be seen in Figure 6d, the Fe_3_O_4_/Ag/RGO-coated cotton fabric reflects only a very small amount (less than 0.5 dB) of electromagnetic waves, while the vast majority are absorbed by the material. This is attributed to the combined loading of Ag and RGO, which improves the electrical conductivity of the cotton fabric, while the loading of Fe_3_O_4_ endows the fabric with magnetic properties and increases its magnetic permeability. Additionally, both Fe_3_O_4_ and Ag are excellent wave-absorbing materials, so the absorption of electromagnetic waves accounts for more than 99% after combination. Absorbing electromagnetic waves rather than reflecting them avoids secondary electromagnetic pollution, meeting the current demands for electromagnetic shielding materials. Therefore, the Fe_3_O_4_/Ag/RGO-coated cotton fabric is an excellent wave-absorbing material.

To highlight the performance advantages of our Fe_3_O_4_/Ag/RGO-coated cotton fabric, its electromagnetic shielding effectiveness with several recently reported cotton fabric-based EMI shielding materials [19,20,21,22] is compared, as shown in Table 1. Most existing cotton-based shields exhibit EMI SE values below 45 dB. In contrast, the Fe_3_O_4_/Ag/RGO-coated cotton fabric achieves an EMI SE exceeding 45 dB across the entire 1–18 GHz range, with a maximum of 55 dB. This represents a significant improvement over the compared literature. Furthermore, unlike most reflective-dominant cotton shields, the Fe_3_O_4_/Ag/RGO-coated fabric achieves an absorption-dominated shielding mechanism (>99% absorption), which effectively avoids secondary electromagnetic pollution. The superior performance of our fabric is primarily due to the synergistic combination of three functional layers: the inner RGO layer establishes a conductive network; the intermediate Ag nanoparticles provide high electrical conductivity and dielectric loss; and the outer Fe_3_O_4_ layer introduces magnetic loss mechanisms. This triple-layer hierarchical structure not only enhances total shielding effectiveness but also promotes impedance matching, thereby facilitating wave absorption rather than reflection. Consequently, the obtained fabric achieves a rare combination of high EMI SE (>45 dB) and absorption-dominated shielding (>99%), making it highly suitable for applications where secondary electromagnetic pollution must be minimized, such as in stealth technology and military protective clothing.

The Fe_3_O_4_/Ag/RGO-coated cotton fabric offers distinct advantages in terms of flexibility after folding (Figure 1) compared with many recently developed EMI shielding materials [23,24,25]. Although numerous high-performance shields have been reported, such as MXene-based films [23], carbon nanotube aerogels [24], and metal-based materials [25], most of these materials are either rigid, free-standing films or require rigid substrates that cannot conform to curved surfaces or withstand repeated bending. In contrast, the obtained fabric is based on a commercial cotton textile substrate, which inherently provides excellent flexibility, drapeability, and breathability. The triple-layer coating (Fe_3_O_4_/Ag/RGO) is deposited uniformly onto individual cotton fibers without compromising the intrinsic softness or mechanical integrity of the fabric. Therefore, the Fe_3_O_4_/Ag/RGO-coated fabric is uniquely positioned for wearable and flexible applications.

**Figure 7:**
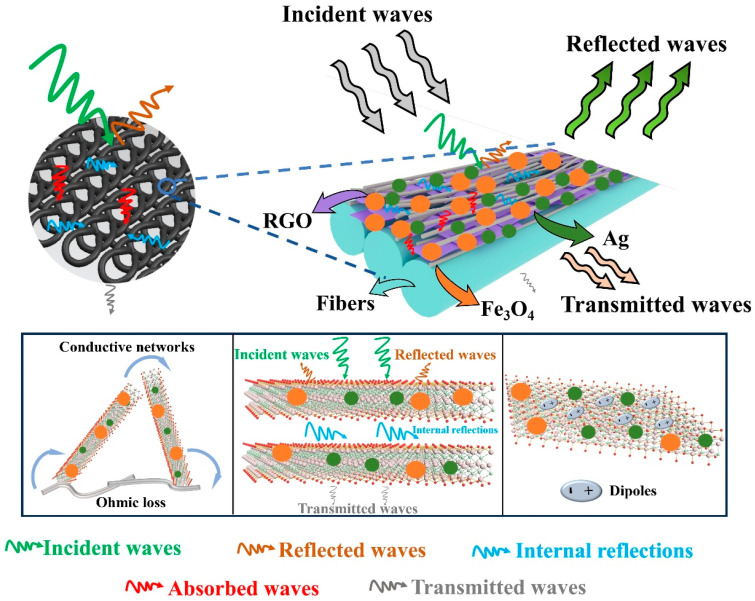
Electromagnetic wave shielding mechanism of Fe_3_O_4_/Ag/RGO-coated cotton fabrics.

The electromagnetic interference (EMI) shielding mechanism of the Fe_3_O_4_/Ag/RGO triple-layer-coated cotton fabric is illustrated in Figure 7. The outer layer consists of Fe_3_O_4_ nanoparticles, which primarily contribute to magnetic loss. When incident waves strike the fabric surface, a portion of the electromagnetic waves is immediately reflected due to the impedance mismatch between air and the Fe_3_O_4_ layer. The remaining waves penetrate the outer layer and encounter the intermediate Ag nanoparticle layer. This layer forms a highly conductive network, inducing strong ohmic loss and dipolar polarization, which effectively attenuates the electromagnetic energy through absorption and multiple internal reflections. The transmitted waves that pass through the Ag layer then reach the bottom RGO nanosheet layer. RGO provides additional conductive pathways and a large specific surface area, further promoting ohmic loss and interfacial polarization [13]. Meanwhile, electromagnetic waves that are not fully absorbed are reflected back and forth between the conductive Ag network and the RGO layer, creating multiple reflections that extend the propagation path and enhance energy dissipation. As a result, the triple-layer structure synergistically integrates reflection, absorption, and multiple internal reflections, leading to superior EMI shielding effectiveness dominated by absorption mechanisms. All in all, the Ag intermediate layer provides ultra-high electrical conductivity, while the Fe_3_O_4_ outer layer contributes magnetic permeability. Critically, Fe_3_O_4_ enables absorption-dominated shielding (>99% absorption) by improving impedance matching between air and the highly conductive Ag layer, a function that electrical conductivity alone cannot achieve. In conclusion, three mechanisms dominate the electromagnetic wave attenuation among the multiple loss mechanisms in the Fe_3_O_4_/Ag/RGO triple-layer composite: ohmic loss from the highly conductive Ag network; interfacial polarization at the Fe_3_O_4_/Ag and Ag/RGO heterojunctions generates charge accumulation and relaxation losses that convert electromagnetic energy into heat [26,27]; and magnetic loss from Fe_3_O_4_ (natural resonance and eddy current loss) critically enables impedance matching. The Ag/RGO bilayer lacks Fe_3_O_4_, resulting in reflection-dominated shielding (SE_R_ > SE_A_). After Fe_3_O_4_ deposition, the dominant mechanism shifts to absorption-dominated (>99% absorption), with interfacial polarization and magnetic loss playing enabling roles that reflection alone cannot achieve. A key advantage of the obtained fabric is its absorption-dominated shielding, which fundamentally differs from reflective metal-based shields that cause secondary electromagnetic pollution. This makes Fe_3_O_4_/Ag/RGO triple-layer-coated fabric particularly suitable for military stealth applications, where reflected signals can reveal personnel or equipment, as well as for wearable devices requiring breathability and flexibility.

## 4. Conclusions

In this work, Fe_3_O_4_/Ag/RGO triple-layer nanocomposites were successfully coated onto the cotton fabric. The Fe_3_O_4_/Ag/RGO-coated cotton fabric exhibits a resistance of 1.68 Ω/sq, demonstrating excellent conductive performance. Among the composite-coated cotton fabrics, the Fe_3_O_4_/Ag/RGO-coated cotton fabric exhibited the best electromagnetic shielding performance due to its high dielectric loss and magnetic loss, achieving electromagnetic shielding effectiveness exceeding 45 dB across the entire 1–18 GHz frequency range and reaching 55 dB at 16 GHz. Furthermore, the Fe_3_O_4_/Ag/RGO-coated cotton fabric reflected only a very small amount (less than 0.5 dB) of electromagnetic waves, with the vast majority being absorbed by the material. The absorption of electromagnetic waves accounted for more than 99%. Therefore, the Fe_3_O_4_/Ag/RGO-coated cotton fabric is an excellent electromagnetic wave-absorbing material. The triple-layer design synergistically combines high electrical conductivity (from Ag) with magnetic permeability (from Fe_3_O_4_), where Fe_3_O_4_ plays the unique role of enabling absorption-dominated shielding through impedance matching. The microwave-assisted fabrication method used in this work is rapid, energy-efficient, and scalable for industrial production, offering a practical pathway for commercial manufacturing of high-performance EMI shielding textiles. Compared with other high-performance EMI shielding materials, the obtained fabric offers a unique combination of textile-grade flexibility, low-cost commercial cotton substrate, and simple microwave-assisted fabrication, making it a practical and scalable solution for wearable and military applications.

## Figures and Tables

**Table 1:** Comparison of the EMI shielding effectiveness of cotton fabric-based materials reported in the recent literature.

Materials	EMI SE (dB)	Frequency Range	Reference
Fe_3_O_4_/Ag/RGO/cotton	>45(up to 55 dB)	1–18 GHz	This work (trilayer)
Ti_3_C_2_T_x_ MXene/cotton	up to 48.9	2–18 GHz	[19]
Fe_3_O_4_/PPy/textile	40–47	8.2–12.4 GHz	[20]
PPy/cotton	28.2	8.2–12.4 GHz (X-band)	[21]
PEDOT:PSS/cotton	up to 46.61 dB	8.2–12.4 GHz (X-band)	[22]

## Data Availability

The original contributions presented in this study are included in the article. Further inquiries can be directed to the corresponding author.
